# Virtual memory T cells develop and mediate bystander protective immunity in an IL-15-dependent manner

**DOI:** 10.1038/ncomms11291

**Published:** 2016-04-21

**Authors:** Jason T. White, Eric W. Cross, Matthew A. Burchill, Thomas Danhorn, Martin D. McCarter, Hugo R. Rosen, Brian O'Connor, Ross M. Kedl

**Affiliations:** 1 Department of Immunology and Microbiology, University of Colorado Denver at Anschutz Medical Campus, School of Medicine, Aurora, Colorado 80045, USA; 2 Department of Medicine and Division of Gastroenterology and Hepatology, University of Colorado Denver at Anschutz Medical Campus, School of Medicine, Aurora, Colorado 80045, USA; 3 Department of Biomedical Research, National Jewish Health, Denver, Colorado 80206, USA; 4 Department of Surgery, University of Colorado Denver at Anschutz Medical Campus, School of Medicine, Aurora, Colorado 80045, USA

## Abstract

Virtual memory cells (VM) are an antigen-specific, memory phenotype CD8 T-cell subset found in lymphoreplete, unchallenged mice. Previous studies indicated that VM cells were the result of homeostatic proliferation (HP) resembling the proliferation observed in a lymphopenic environment. Here we demonstrate that HP is ongoing in lymphoreplete mice, the degree of which is dictated by the number of naive CD8 T cells with a sufficiently high affinity for self-antigen interacting with peripheral IL-15. VM cell transcriptional profiles suggest a capacity to mediate protective immunity via antigen non-specific bystander killing, a function we show is dependent on IL-15. Finally, we show a VM-like population of human cells that accumulate with age and traffic to the liver, displaying phenotypic and functional attributes consistent with the bystander protective functions of VM cells identified in the mouse. These data identify developmental and functional attributes of VM cells, including their likely role in protective immunity.

## Abstract (web summary)

Virtual memory T cells are CD8 T cells with memory phenotype present in unimmunized mice. Here the authors show that these cells have higher affinity for self-antigen, depend on IL-15 for proliferation and antigen-non-specific cytotoxicity in mice, and that a similar population exists in humans.

## Graphical abstract

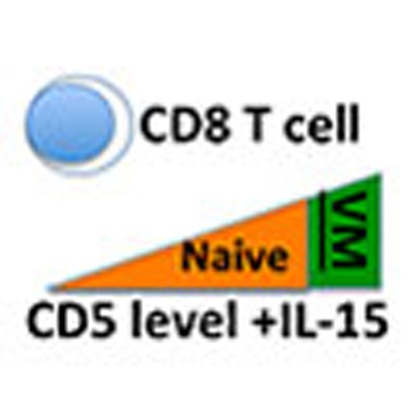

Besides the numerous memory T-cell subsets that arise following antigenic challenge, it is now clear that memory phenotype (MP) CD8 T cells can be found in all mice regardless of prior pathogen exposure. Many of these MP subsets, such as CD8 intraepithelial lymphocytes or innate CD8s, have a well-described development that depends on thymic signalling^1^,^2^,^3^. Much less is known about the development of another MP subset, CD44hi/CD122hi/CD49dlo CD8 cells, which is specific for nominal antigen but present in antigen-inexperienced mice. While we and our collaborators coined the term ‘virtual memory' (VM) to described this cellular subset, the presence of MP cells in the unprimed host had been long been known^4^, but were largely assumed to represent cells that had undergone antigen-mediated expansion to microbiome- or food-associated antigens. As a result, the repertoire of these MP cells was not expected to possess any cells specific to nominal/novel antigens except as a result of cross-reactivity to related antigens. In our original description of VM cells, we demonstrated their development depended on homeostatic, not antigenic, cues in the environment, and that within their ranks were included T cells specific to nominal antigens^5^. Since then, we and others have shown that VM cells arise in the periphery^6^ in a PLZF/IL-4/NKT cell-independent, but interleukin (IL)-15-dependent, manner^7^, once developed they can respond vigorously to cytokines such as IL-4 (ref. 8) and type I interferon (IFN)^9^, and that they accumulate in the aged host^10^. As with memory cells in general, VM cells make IFNγ in response to stimulation with IL-12 and IL-18 (ref. 5), and, similar to homeostatic proliferation (HP) memory T cells derived from a lymphopenic environment, are efficient in mediating a protective response against a cognate antigen-expressing pathogen^7^,^11^. Considering that VM cells make up 15–25% of the unprimed CD8 pool (in unmanipulated B6 mice), functional benefits commensurate with their prevalence in the repertoire have yet to be clarified.

The identification of VM cells contributes to the growing recognition that, much similar to the antigen-experienced repertoire of memory T cells, the antigen-inexperienced repertoire displays substantial heterogeneity. More recent evidence shows that the naive (CD44lo) CD8 pool in the periphery has different functionality dependent upon selection signals received in the thymus. Indeed, data have shown that T cells emerging from the thymus with higher affinity for self-antigens (expressing high levels of CD5 [CD5hi]) display a distinct advantage in becoming engaged in both homeostatic and antigen-mediated response when compared with their CD5lo counterparts^12^,^13^. Recent data examining the gene expression profile of CD5hi and CD5lo naive T cells suggests that CD5hi cells are transcriptionally poised to engage both proliferative and effector functions far more rapidly than CD5lo cells of the same specificity^14^. While these studies are informative as to the naive T-cell response to antigen in an inflammatory setting, the cues by which a naive phenotype T cell within the periphery integrates tonic and cytokine signals in a non-lymphopenic environment to become a member of the VM pool are still poorly defined. Furthermore, VM cells have thus far only been studied in mice, although putative human analogues have been suggested^15^,^16^.

In the present work, we now provide conclusive evidence that VM cell development is a natural consequence of the heterogeneity of the naive CD8 T-cell pool. We show that VM cells are not only derived from cells with increased affinity for self-antigens but they also have higher affinity for their cognate antigens than naive phenotype T cells of the same specificity. As has been described for naive T cells during an antigen-specific response, naive CD8 T cells of the highest self-affinity (as measured by CD5) are the most likely ones to undergo IL-15-dependent HP that leads to VM development in the lymphoreplete host. RNA sequencing (RNAseq) comparing naive and VM subsets confirms recently published data on naive T cells^14^ and further connects VM cell development to the CD5hi naive repertoire. Differences between CD5hi naive, VM and antigen-experienced memory cells in their expression of NKG2D and GzmB further predicted a role for VM cells in mediating bystander immune protection^17^. Surprisingly, in the absence of IL-15, VM expression of granzyme B and NKG2D was nearly ablated, as was their capacity to mediate bystander protection. These data give a new functional role for VM cells during exposure to pathogens independent of antigen specificity as well as a new role for IL-15 during the primary response. Finally, we show that a previously identified VM-like subset in humans traffics extensively to the liver, accumulates with age and collectively displays the phenotypic and functional traits identified in the mouse.

## Results

### CD5 expression correlates with increase VM development

**Figure 1:**
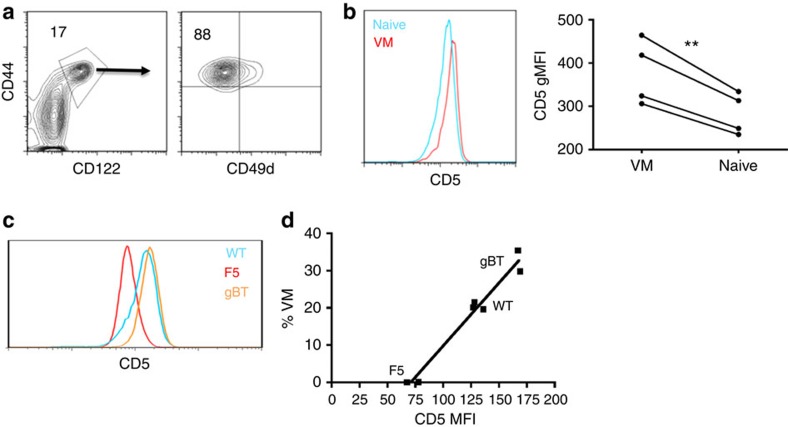
VM cells resemble cells that have undergone HP in both integrin expression and affinity. (**a**) Flow cytometry on splenocytes isolated from unmanipulated WT mice. Gating on CD44hi/CD122hi CD8 T cells (left, percentage of CD8 above gate). Gated population visualized by CD44/CD49d (right, percentage of VM cells [CD44hi, CD49dlo] above population). (**b**) Naive (CD44lo) and VM (CD44hi, CD49dlo) CD8 T cells from the same mouse stained for CD5. Representative histogram overlay of the two populations (left), gMFI comparison (right; **P<0.01, paired *t*-test, representative of two experiments of *n*=4 per group and *n*=3 per group). (**c**,**d**) Naive (CD44lo) CD8 T-cell populations from polyclonal and WT mice were stained for CD5. (**c**) Representative flow histogram overlay of naive CD5 distributions for WT (blue), F5xRag^−/−^ (red) and gBTxRag^−/−^ (orange) (one mouse per histogram; representative of three experiments). (**d**) gMFI of naive populations graphed against per cent VM cells found in each mouse (linear regression, *r*^2^: 0.97, *P*<0.0001; representative of two experiments with at least *n*=2 per group).

VM cells are CD8+CD44hi,CD49dlo, allowing their reliable separation from naive (CD44lo) and antigen-experienced memory cells (CD44hi,CD49dhi)^5^ (Fig. 1a). Other than the fact that these cells arise in the periphery and are dependent on IL-15, little else was known about VM development. A non-stochastic mechanism was initially suggested by the difference in CD5 expression between VM and naive cells. Expression of CD5 reflects a T cell's affinity for the self-ligands on which it was selected in the thymus^18^. Numerous labs have reported that naive T cells expressing higher CD5 show increased proliferation in response to lymphopenia^13^,^19^ or antigenic stimulation^12^,^14^ relative to their CD5lo counterparts. Analysis of bulk naive and VM CD8+ T cells within an unmanipulated B6 host for CD5 expression levels revealed a statistically significant increased expression of CD5 on all VM cells relative to naive (Fig. 1b). Further, CD5^−/−^ hosts showed an increase in VM cells relative to wild-type (WT; Supplementary Fig. 1), allowing us to rule out any role for direct CD5 signalling in VM cell formation. Considering T cells developing in the CD5^−/−^ host experience stronger T cell receptor (TCR) stimulation during selection^20^, these data in conjunction with Fig. 1b are consistent with the conclusion that the VM pool was likely derived from cells that had been selected with high affinity/avidity on self-antigens in the thymus.

We previously showed that VM cells are present even in TCR transgenic crossed onto Rag-deficient hosts (Rag^−/−^; ref. 5), a result that supported the conclusion that VM cells develop independently of their encounter with cognate antigens. In comparing VM cells from WT B6 mice with T cells from either the gBT TCR transgenic (specific for a peptide from herpes simplex virus (HSV) glycoprotein B) or the F5 TCR transgenic (specific for a peptide from influenza nucleoprotein (NP)), each crossed to the Rag^−/−^ (gBTxRag, F5xRag), we noted a direct correlation between CD5 expression and the frequency of VM cells. T cells from the gBT and F5 TCR transgenics have higher and lower CD5 expression levels, respectively, as compared with the polyclonal repertoire in WT B6 (Fig. 1c)^19^. When plotted against the percentage of T cells in these hosts that are of VM phenotype, we obtained a near straight-line relationship (Fig. 1d). This was further evidence that the affinity of a T cell for selecting ligands in the thymus (as gauged by CD5 expression) may be a predictor of a cell's propensity to convert to a VM phenotype.

### VM derive from naive cells with high self-affinity

**Figure 2:**
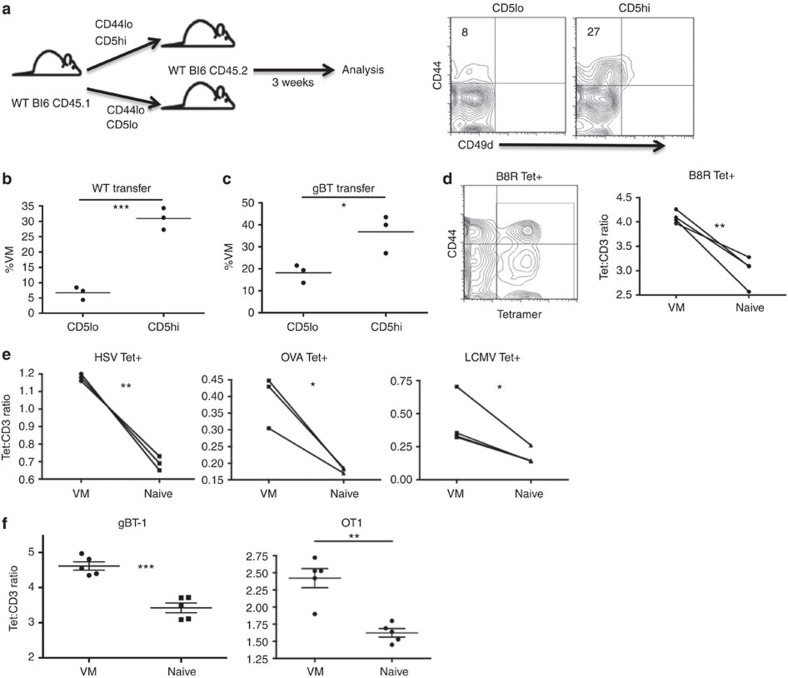
CD5 expression is a predictor of a naive cell's propensity to become VM. (**a**) Naive (CD44lo), CD5hi (the 20% highest CD5 expressers) or CD5lo (the 20% lowest CD5 expressers) CD8 T cells were sorted out of unmanipulated WT mice, and 4.0 × 10^5^ of each population were adoptively transferred into congenic WT recipients, which were left undisturbed for 3 weeks (left). Recipients were then killed and splenocytes were analysed for transferred cells changing phenotype (right, per cent VM in quadrant). (**b**,**c**) Comparisons between WT mice receiving WT (**b**) or gBTxRag^−/−^ (**c**) transferred cells (*P<0.05, ***P<0.001, *t*-test; *n*=3 per group). (**d**,**e**) Tetramer staining and pulldown of CD8s specific for foreign cognate antigen in WT mice. Representative contour flow plot of CD44/tetramer staining for B8R tetramer (left), overlay of pseudocolour dot plots for CD3 and tetramer staining in naive (blue) and VM (red) CD8 T cells. Comparison within mice of tetramer:CD3 gMFI staining on individual tetramer+ (Tet+) cells (**d**, B8R [TSYKFESV]; **e**, HSV [SSIEFARL], OVA [SIINFEKL], LCMV [KAVYNFATC]; *P<0.05, **P<0.01, paired *t*-test, representative of 2–4 experiments with *n*=3 per group). (**f**) PBMCs from gBT-1 and OTI TCR transgenic mice were stained with the HSV and OVA MHCI tetramers, respectively, as described above. VM and naive populations were compared within mice for Tet:CD3 ratios (**P<0.01, ***P<0.001, paired *t*-test; *n*=5 per group, error bars represent s.e.m.).

It was conceivable that VMs had increased CD5 levels as a result of cellular activation, as some data have suggested^20^, giving the appearance of having increased affinity for self-antigen. To establish a causal relationship between CD5 expression and propensity for VM conversion, we isolated CD5hi and CD5loCD44lo polyclonal CD8+ T cells from unmanipulated B6 WT mice and adoptively transferred them into separate congenic, lymphoreplete WT mice. After 3 weeks, the donor cells were isolated and phenotyped. As anticipated, a significantly greater percentage of the transferred CD44loCD5hi cells had acquired a VM phenotype (CD44hi, CD49dlo) as compared with the transferred CD5lo cells (Fig. 2a,b), in agreement with recent data from Jameson and colleagues^14^. The few VM cells derived from the CD5lo transfers maintained a lower expression of CD5 compared with CD5hi-derived VM converts (Supplementary Fig. 2). Although we cannot discount the possibility of minor fluctuations in CD5 levels, these data indicate that CD5 expression is not markedly upregulated during the process of VM conversion. Regardless of minor changes in CD5 expression, these data are consistent with a causal relationship between the CD5 expression profile of a naive CD8+ T cell and its propensity to convert into VM cell within the periphery.

CD5hi naive T cells have also shown an increased response to antigenic challenge^12^,^14^,^21^. The preferential responsiveness of CD5hi over CD5lo T cells can be observed even between T cells with identical TCRs, effectively separating the responsiveness of the T cell from its affinity for cognate antigen^12^,^14^. Using T cells from the gBTxRag host, we examined whether the conversion of CD5hi T cells to VM could similarly occur irrespective of TCR affinity. Similar to the results with bulk naive T cells, transferred CD44loCD5hi gBT cells were significantly more likely to convert to VM phenotype compared with transferred CD44loCD5lo gBT cells (Fig. 2c).

Previous studies have looked at the relationship between CD5 levels and affinity for foreign antigen, but none included VM cells^12^,^14^,^21^. As such, we used a tetramer staining and magnetic enrichment method^5^ to compare naive and VM cells' affinity for the vaccinia virus epitope, B8R. Using flow analysis software, we divided the tetramer mean fluorescent intensity (MFI) for each event against its own CD3 MFI. This method corrects for any reduced tetramer staining that is a result of reduced TCR levels and produces a quantification of relative affinity of the TCR for antigen^22^. VM cells demonstrated a significantly higher apparent affinity for cognate antigen as compared with naive phenotype T cells of the same specificity (Fig. 2d). This was not an artefact of B8R-specific T cells, as this pattern held true for T cells specific for Ova, HSVgB and LCMV GP33 (Fig. 2e).

These data supported a possible connection between a T cell's affinity for self-ligands and its affinity for cognate antigens. However, naive and memory T cells are known to interact with multimeric peptide:major histocompatibility complex (MHC) with very different avidities due to changes in cell membrane composition and not TCR affinity^23^. Again, analysing T cells from the TCR transgenics, we observed that VM cells with the same TCR as their naive counterparts maintained an increased tet:CD3 ratio (Fig. 2f), consistent with their having an overall higher avidity for peptide:MHC. Although it is still conceivable that VM cells in the polyclonal repertoire also have an increased affinity for cognate antigens, this parameter is obscured by an increase in avidity endowed by their conversion to MP. Overall, these data continue to indicate that the VM pool is not derived stochastically from the entire naive T-cell repertoire. Rather, it is derived from the subset of naive T cells that were selected on higher affinity self-ligands, ultimately producing MP cells with increased binding to multimeric cognate peptide:MHC as well.

### IL-15 availability dictates VM cell conversion

**Figure 3:**
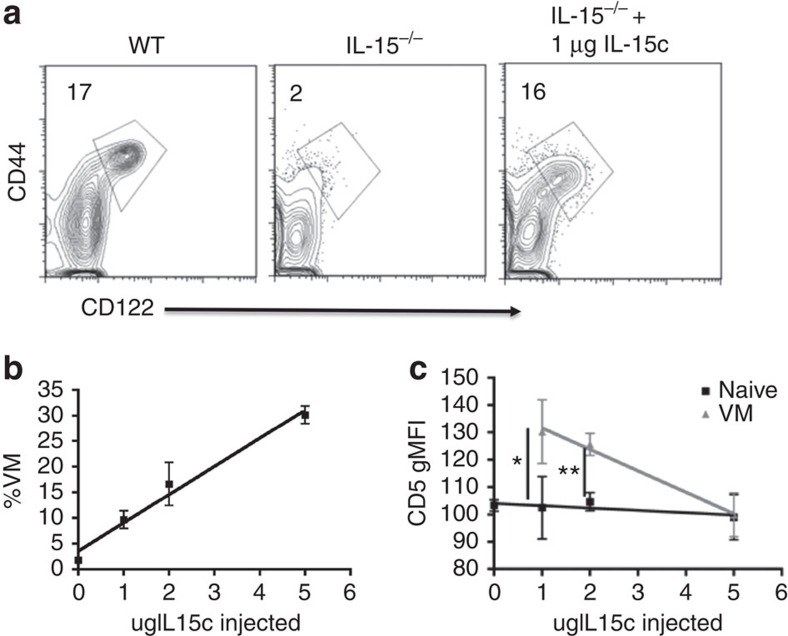
Availability of IL-15 dictates both the size and CD5 composition of the VM population. (**a**) Percentage of VM (CD44hi, CD122hi) cells found in WT (left), IL-15^−/−^ (middle) and IL-15^−/−^ 4 days after IL-15 complex injections (right, per cent VM above gate). (**b**,**c**) IL-15 complexes were injected intravenously and given 4 days to interact with the hematopoietic system. Splenocytes were then analysed for their percentage of VM cells as a function of IL-15 complexes injected (**b**, linear regression, *r*^2^=0.98, *P*=0.01; representative of two experiments with *n*=3 per group), and the gMFI of CD5 in the VM and naive pools (**c**, *P<0.05, **P<0.01, *t*-test, representative of three experiments with *n*=3 per group, error bars represent s.e.m.).

Previous reports indicated that CD5 expression also predicts the responsiveness of CD8+ T cells to undergo HP in response to IL-2/15 under non-lymphopenic conditions^19^. This fit well with our data, as we and others have already established that VM cells form in the periphery as a result of IL-15-dependent HP^7^,^11^ (Fig. 3a). Combined, these data suggest that the size of the VM pool is dictated both by the affinity of the T-cell repertoire as well as the availability of IL-15. To test this prediction, we injected the IL-15^−/−^ host with IL-15/Rα complexes^24^ (IL-15c). As shown previously, an IL-15^−/−^ host has no VM cells (Fig. 3a). Four days post-IL-15c injection, there is a dose-dependent increase in VM cells (Fig. 3b). There was also an inverse relationship between the CD5 geometric mean fluorescence intensity (gMFI) of the VM cells generated and the dose of IL-15c injected (Fig. 3c). This was not because IL-15 stimulation caused reduced CD5 expression, as OT1 VM cells transferred into a naive recipient maintained the same levels of CD5 with or without subsequent IL-15c injection (Supplementary Fig. 3). Thus, as more IL-15 becomes available, T cells with lower affinity for self-antigens convert to VM phenotype, such that the gMFI of CD5 on the VM cells becomes indistinguishable from that of the naive phenotype cells (Fig. 3c). Collectively, these data (Figs 1, 2, 3) indicate that the pool of VM phenotype cells arises as a result of naive T cells with the high affinity for self-antigens responding to limiting amounts of IL-15 trans-presented in the periphery.

### RNA profile shows homeostatic and inflammatory sensitivity

**Figure 4:**
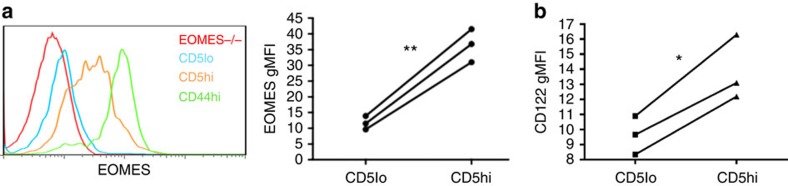
RNAseq highlights differences in cytokine responsiveness between CD8 T naive CD5hi/lo and VM cells. (**a**) The T-cell population of a WT mouse was stained for eomesodermin expression. The representative histogram overlay of the different populations is given (left), as well as a comparison between naive (CD44lo) CD5lo/CD5hi cells (**P<0.01, paired *t*-test, representative of two experiments with *n*=3 per group). (**b**) Naive CD8 T cells were also stained for IL-2/15Rβ (CD122) expression (*P<0.05, paired *t*-test, representative of two experiments with *n*=3 per group).

The transcription factor Eomes and its induction of the IL-2/15Rβ CD122 are essential for the CD8+ T-cell response to IL-15 (ref. 25). These gene products are also elevated in VM cells relative to the total naive pool^7^,^11^. We observed a substantial difference between CD44loCD5lo and CD44loCD5hi T cells in their expression of these proteins, with essentially all Eomes and CD122 expression contained within the CD5hi's (Fig. 4a,b). To better examine gene expression, we performed RNAseq by comparing the total RNA expression in CD5loCD44lo, CD5hiCD44lo and VM cells (Supplementary Data 1). By comparing the CD5lo and CD5hi naive T-cell profiles, we found ∼600 genes that were differentially expressed (Supplementary Data 2); a gene set that was in good agreement with recently published RNAseq data from Fulton *et al*.^14^ Kyoto encyclopedia of genes and genomes (KEGG) analysis revealed ‘cytokine/cytokine receptor interaction' as the dominant cellular pathway associated with these genetic differences (Table 1). Gene ontology (GO) analysis identified 10 cellular functions as significantly increased (Table 2); the majority of which related to the regulation of life and death pathways. Curiously, other than the chemokine receptors *Cxcr3* and *Ifngr1*, genes that might predict increased responsiveness to the inflammatory milieu (for example, *Cxcr5*, *Il18rap*, *Il12rb1*, *Il18r1*, *Stat4* and *Ccr2*) only showed substantial changes in expression (>15 transcripts per million) after differentiation to VM cells (Table 1). In contrast, the greatest increases were seen in cytokine genes associated with homeostatic cytokine responsiveness (*Il2rb*, *Eomes* and *Il4r*).

In comparing gene expression between CD5hi T cells and VM cells, substantially more genes (1909; Supplementary Data 3) and gene ontology pathways (Table 2) showed differential expression, although ‘cytokine signalling' continued to be the major associated KEGG pathway (Table 1). In addition to even greater increases in the genes necessary for response to homeostatic cytokines IL-2/7/15 (*Il2rb*, *Il4r*, *Il7r*, *Eomes* and *Tbx21*), we also observed robust increases in genes involved in producing and/or sensing the pro-inflammatory milieu (*Ccl3/4/5*, *Il18r1*, *Il18rap*, *Il12r1*, *Stat4* and tumor necrosis factor *Tnf*), chemotaxis (*Ccr2/5*, *Cxcr3/5* and *Xcl1*), sensing costimulatory ligands (*Tnfsf14* and *Tnfrsf9*) and effector molecules associated with target recognition (*Nkg2d*) and cell killing (*Grzb*, *Ifng* and *Fasl*) (Table 1). These molecules indicate that the conversion of CD5hi naive T cells into VM cells results in their increased sensitivity to both homeostatic and inflammatory cues, consistent with the functional attributes of VM cells previously described^5^,^7^,^11^.

### VM cells effect bystander protection

Besides showing strong upregulation of cytokine and chemokine signalling networks, the RNAseq data also showed that VM cells potently express molecules (IL-12R, IL-18R, IFNγ, GrzB and NKG2D) known to enhance innate-like effector functions, which have been connected to so-called ‘bystander protection' of memory CD8+ T cells^17^. Increased expression of IL-12R and IL-18R facilitate the VM cell production of IFNγ even in the absence of TCR stimulation^5^, and GrzB and NKG2D expression can mediate antigen-independent killing of infected target cells by antigen-experienced memory T cells^17^. We therefore speculated that VM cells may be involved in bystander killing.

**Figure 5:**
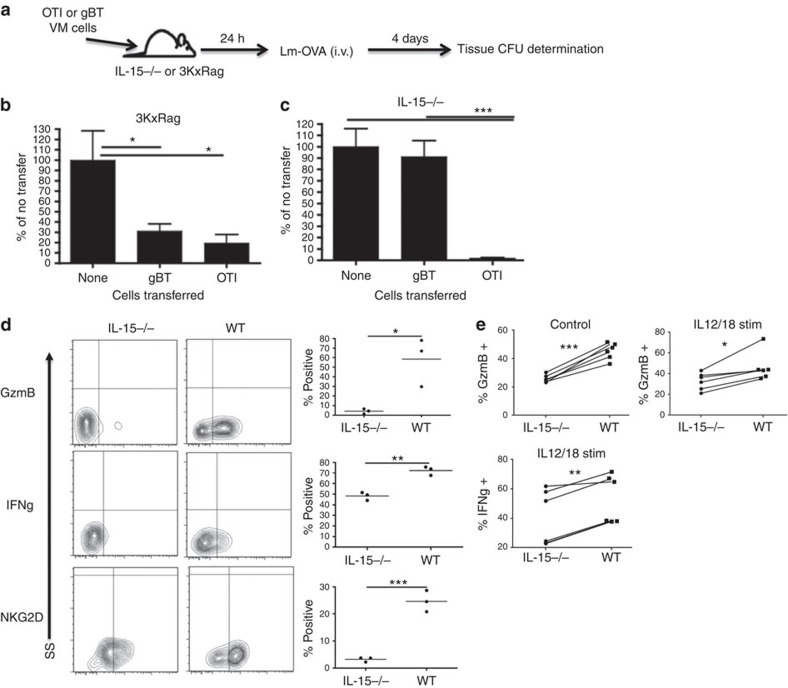
VM cells, being similar in phenotype to previously reported bystander cells, mediate their effects in a bystander-like manner. (**a**) OTI and gBTxRag^−/−^ mice were sorted for VM cells and subsequently injected into 3KxRag^−/−^ (**b**) or IL-15^−/−^ (**c**) mice (2.0 × 10^5^ cells per mouse). One day after transfer, mice were challenged with Lm-OVA via tail vein (1.0 × 10^5^ CFU per mouse for 3KxRag^−/−^ and 2.5 × 10^3^ CFU per mouse for IL-15KO). Four days later, mice were killed and spleens were processed for CFU (**b**,**c**, *P<0.05, ***P<0.001, one-way analysis of variance, data combined from two experiments with *n*=3 per group, error bars represent s.e.m.). (**d**) Mice from the gBTxRag^−/−^ transfer conditions (into WT or IL-15^−/−^ mice) were injected with Brefeldin A before killing, and the transferred cells were subsequently stained for effector molecule expression (*P<0.05, **P<0.01, ***P<0.001, *t*-test, representative of two experiments with *n*=3 per group). (**e**) Congenically marked WT T cells were incubated with WT or IL-15^−/−^ splenocytes for 24 hours with (IL12/18 stim) or without (control) IL12/18 stimulation, then stained for effector molecule expression (*P<0.05, **P<0.01, ***P<0.001, paired *t*-test, data combined from two experiments with *n*=3 per group).

Given previously published work on listeria monocytogenes (LM) infection as a model for CD8 bystander killing^17^, we used an LM-ova protection assay with the adoptive transfer of VM cells isolated from antigen-specific (OT1) or antigen-irrelevant (gBT-1) donors (Fig. 5a). VM cells typically comprise anywhere from 15 to 25% of CD8+ T cells in an unmanipulated B6 host, at least some of which are specific for nominal antigens such as ovalbumin^5^, the presence of which confounds attempts to measure a non-antigen-specific challenge. We therefore utilized two different hosts as VM transfer recipients (3KxRag and IL-15^−/−^), both chosen because of their lack of endogenous VM cells. First, we utilized a Rag^−/−^ crossed to the 3K CD4+ TCR transgenic host (3KxRag)^26^. This host has no VM cells because it lacks all CD8+ T cells and there is no VM equivalent in the CD4+ T-cell compartment^5^,^7^. Importantly, however, the presence of the irrelevant CD4+ T cells prevents canonical lymphopenia-induced T-cell expansion of any transferred T cells. VM cells were sorted from either OT1 or gBT-1 transgenic hosts, transferred into Rag3K mice, and 24 h later the recipients were challenged with LM-ova. Four days after challenge, the mice were killed and splenic bacterial counts were determined. As previously published^7^,^11^, OTI cells protected against LM-ova challenge, mediating a substantial reduction in splenic bacterial CFUs. gBT cells also protected against LM-ova challenge to a magnitude that was, surprisingly, indistinguishable from that mediated by OTI cells (Fig. 5b). Thus, VM cells are capable of mediating potent immunological protection against bacterial challenge even in the absence of their cognate antigen.

### IL-15 is required for VM bystander protection

In addition to the 3KxRag recipient above, we performed similar bystander protection experiments using IL-15^−/−^ mice as recipients because these hosts also lack all VM cells (Fig. 3). In contrast to the results in the 3KxRag hosts, only the antigen-specific VM cell transfers (OTI) showed any protective effect in this system (Fig. 5c). Although the IL-15^−/−^ host is also devoid of both natural killer (NK) and natural killer T (NKT) cells, previously published data showed that protection against LM challenge does not require either of these cell types^27^,^28^,^29^. The lack of bystander protection in the IL-15^−/−^ therefore indicated that IL-15 was necessary for VM cells to mediate this function. To examine the effect of IL-15 on T-cell effector functions under conditions of bystander killing/protection, we performed adoptive transfer of gBT VM cells into WT and IL-15^−/−^ hosts and challenged with LM-ova as before. On day 4 post infection, we performed an *in vivo* intracellular cytokine stain (ICCS) on transferred cells, looking at molecules crucial to bystander killing^5^,^17^. Most strikingly, gBT VM cells in the IL-15^−/−^ host showed substantially reduced GrzB, NKG2D and IFNγ expression (Fig. 5d). These differences in GrzB and IFNγ were also observed in VM cells after overnight incubation with WT or IL-15^−/−^ splenocytes (Fig. 5e). Reduced IFNγ and GrzB expression have previously been reported during the primary T-cell response in IL-15^−/−^ mice^30^,^31^. Our data now reveal that IL-15 is also necessary for maximal expression of these effector molecules in the presence or absence of inflammation. We conclude from these data that VM cells are able to mediate robust, non-cognate-antigen bystander protection against bacterial challenge. However, maintaining expression of the effector molecules necessary for this function is dependent on the continued presence of IL-15.

### Trafficking of VM cells

**Figure 6:**
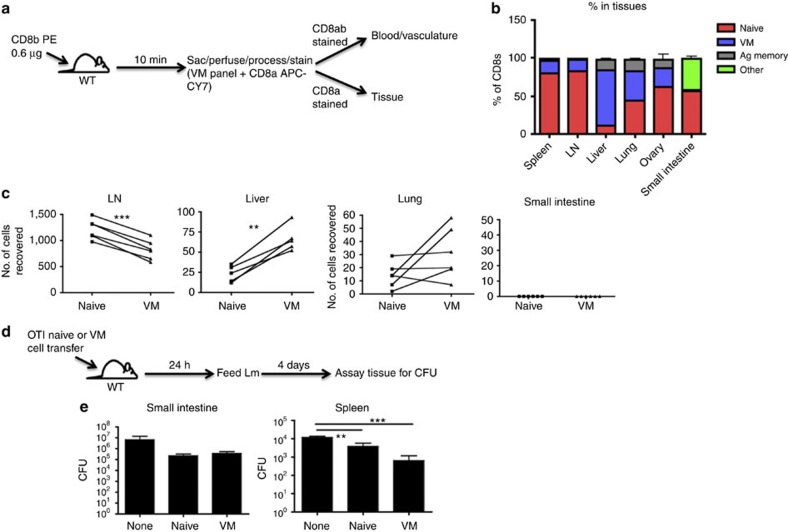
VM cells show a tropism for certain tissues under steady-state and trafficking conditions. (**a**,**b**) WT mice were injected with CD8b antibody that was allowed to briefly circulate. Mice were then killed and stained with an antibody panel to determine naive, VM and antigen-experienced CD8 T cells, and whether the cells were in the vasculature or tissues (**a**). (**b**) Representative bar graphs of T-cell distribution (pooled data from two experiments with *n*=3 per experiment, error bars represent s.e.m.). (**c**) WT splenocytes were sorted into CD8 naive and VM populations, stained with different tracking dyes and co-adoptively transferred into recipient WT congenic mice. Recipients were killed 48 h later and tissues were processed as in **a**, and transferred cell populations were compared (**P<0.01, ***P<0.001, paired *t*-test, data combined from two experiments with *n*=3 per group). (**d**) OTI splenocytes were sorted into naive and VM populations and adoptively transferred into WT mice. One day later, mice were orally challenged with Lm expressing mutant internalin-A, and killed 4 days subsequent to challenge. (**e**) Tissues of mice orally infected were assayed for Lm CFUs (**P<0.01, ***P<0.001, one-way analysis of variance, representative of two experiments with *n*=3 per group, error bars represent s.e.m.).

The unique profile of chemokine receptor and effector molecule expression, (Table 1) and adhesion marker (Table 3) expression in VM cells revealed by the RNAseq data suggested unique tissue trafficking preferences of VM cells as compared with naive. We therefore determined the tissue tropism of VM cells, first focusing on the steady-state distribution of CD8 T cells in unmanipulated, adult (>5 weeks) mice. Using intravenous injection of a labelled anti-CD8b antibody injection to discriminate against vascular and tissue-resident T cells (Fig. 6a)^32^, we isolated various tissues and determined relative percentages of naive, VM and antigen-experienced memory cells by flow cytometry. Perhaps, most striking is the significant proportion of the liver-resident T cells that are VM cells, being generally upwards of 60–70%. Previous studies have described the presence of a considerable pool of memory T cells in the liver^33^, largely assumed to be antigen experienced. Our data presented here indicate that these liver-associated memory cells are overwhelmingly VM cells. In contrast to the liver, the small intestine has essentially no VM cells (Fig. 6b), consistent with their lack of CCR9 and α4β7 expression (Table 3). Adoptive transfer of VM cells showed a similar pattern of tissue tropism, suggesting that VM cells migrate to these sites as opposed to developing *in situ* (Fig. 6c).

### Tissue-specific protection of VM cells

These migratory preferences of VM cells were highly consistent with their protective capacity against LM, a pathogen that replicates primarily in the liver and spleen after a systemic challenge^6^,^7^. To assess whether VM cells were also highly protective against an infectious challenge initiated outside of their tissue trafficking patterns, we utilized a strain of LM-ova expressing modified internalin-A capable of intestinal epithelial cell invasion in the mouse^34^,^35^. Mice were orally challenged with the recombinant LM 1 day after adoptive transfer of naive or VM OTI T cells. Four days after challenge, we measured bacterial counts in both the small intestine and in the spleen (Fig. 6d). The bacterial load in the small intestine was the same after either naive or VM T-cell transfers, indicating that, at least for the gut, VM cells did not display any increased protective capacity relative to naive T cells (Fig. 6d). Bacterial loads in the spleen, however, recapitulated our previous protection assays, showing increased protection in mice transferred with VM cells over that of naive (Fig. 6d). Thus, while VM cells did not provide increased gut-specific protection, they still mediated potent systemic protection against an infection instigated via a gut-specific route.

### Phenotypic similarities in putative human VM cells

**Figure 7:**
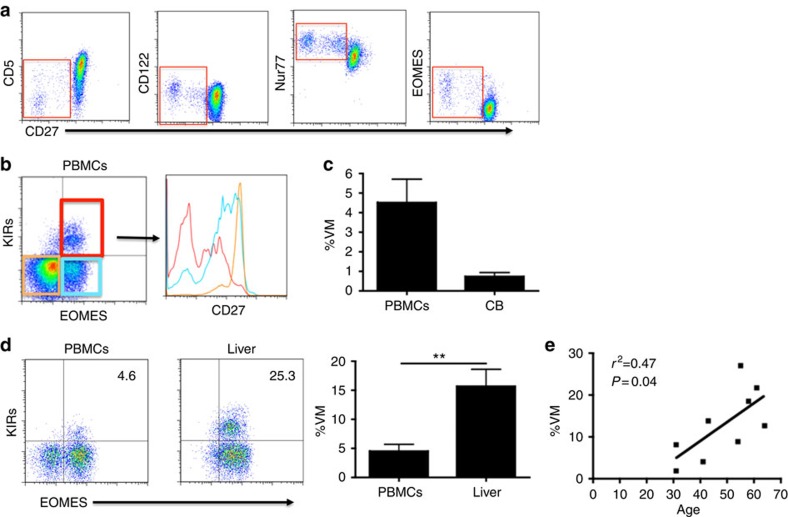
Human subset with VM characteristics. (**a**) Human PBMCs were stained for markers denoting CD8 T-cell interaction with self or IL-15. Representative flow plots of CD8+, CD45RA+ cells are shown. (**b**) Putative human VM memory cells are analysed for their corresponding CD27 levels. (**c**,**d**) Percentage of CD3+/CD8+/CD45RA+/KIR+/EOMES+ (VM) cells are compared between adult PBMC samples and cord blood samples (**c**) or between healthy adult PBMCs (*n*=9, 6 males and 3 females, age range 29–72 (mean 50) years) and healthy liver (*n*=5, 4 males and 1 female, age range 23–56 (mean 40) years; error bars represent s.e.m.; (**d**) **P<0.01, *t*-test). (**e**) Normal splenocytes banked from surgeries (*n*=7 benign pancreatic mass and *n*=2 non-functioning neuroendocrine tumour) were analysed for their populations of VM cells (CD3+/CD8+/CD45RA+/KIR+/EOMES+) and correlated with age (linear regression, *r*^2^=0.47, *P*=0.04).

Thus far, VM cells have been documented only in mice, and it was not clear whether humans produced an equivalent subset. Establishing human equivalents of mouse cellular subsets is often complicated by the disparity in surface marker expression between the two species. Given its centrality in governing mouse VM development, we initially focused on IL-15 responsiveness as a possible identifier of human VM cells. In contrast to CD5 expression in the mouse, human T-cell expression of CD5 is progressively decreased during the process of memory T-cell differentiation. Further, the T cells with the lowest CD5 expression have the highest response to IL-15, express the highest levels of CD122 and are CD45RA^+^/CD27- (refs 36, 37; Fig. 7a). Although these markers place the putative VM cells within a subset previously considered terminally differentiated (terminal effector memory RA+), they bear considerable similarity to mouse VM cells in their higher expression of CD122, responsiveness to IL-15 and expression of both memory and naive phenotypic markers. Furthermore, when analysing Nur77 and Eomes expression in CD45RA+, RO- CD8+ T cells, there was a clear increase in both molecules as CD27 levels drop (Fig. 7a). The Nur77 profile suggests a higher level of basal TCR interactions (higher self-affinity), whereas the Eomes expression suggests a history of exposure to IL-15.

A cell type with a very similar phenotype (CD45RA+CD122hiEomes+) was previously observed in fetal spleen and thymus, although the population was markedly reduced in cord blood and postnatal thymus^38^. Recently, the Eomes+ cells within the CD45RA+ CD8+ T cells were shown to be better identified by co-staining with pan-KIR and NKG2A antibodies^15^. The functional capacity of these CD45RA+Eomes+KIR+NKG2A+ T cells was essentially identical to mouse VM cells: coexpressing Tbet and producing IFNγ in response to IL-12/18 stimulation. Using these markers, we confirmed the observations (Fig. 7b,c) that this subset represents a sizable fraction of total circulating adult (∼5%) and cord blood (∼1%) CD45RA+ T cells.

The presence of these cells in cord blood indicated that it was not necessary for them to be derived from antigen-experienced memory T cells, consistent with their designation as potential VM cells. Besides preferentially trafficking to the liver in mice (Fig. 5), VM cells also accumulate with age^39^. We reasoned that if CD45RA+ KIR+NKG2A+ T cells were VM, then they might display similar trafficking patterns and age distributions in humans. We therefore compared the frequency of CD45RA+ KIR+NKG2A+Eomes+ T cells from normal human liver samples with that observed in the blood. In addition, we examined the frequency of these cells in non-diseased human spleen samples from patients ranging between 30 and 70 years old. As in the mouse, we found the frequency of these cells markedly elevated in the liver (Fig. 7d). Furthermore, we saw a reasonable correlation between age and frequency of KIR+NKG2A+Eomes+ T cells, with older patients achieving frequencies of 20–30% of CD45RA+ T cells in the spleen (Fig. 7e). Collectively, these data suggest that CD45RA+ KIR+NKG2A+Eomes+ T cells may represent the human equivalent of VM cells.

## Discussion

We present above three major findings regarding the development and functional properties of VM cells. First, we show that the development of VM cells is not a stochastic process, but rather proceeds as a result of T cells with high affinity for self-antigens responding to IL-15 in the periphery. The end result is an antigen-inexperienced memory T-cell repertoire skewed towards having a higher affinity for self-antigens and higher avidity for foreign antigens. The fact that all of these events proceed in the lymphoreplete host indicates that HP occurs in the WT environment in the absence of overt lymphopenia. Second, besides having higher affinity for self-antigens, VM cells also express adhesion, chemotactic and effector molecules that facilitate an ‘innate-like' response to inflammatory cues. The combination of these attributes make VM cells ideally suited for mediating both antigen-specific and bystander protective immunity even against infectious challenges that have never previously been encountered. Third, the collective properties of IL-15 sensitivity, apparent affinity for self-antigen, sensitivity to the inflammatory milieu and immediate effector functions are maintained in a unique population of cells that most likely represent the human VM equivalent. These human VM cells additionally reflect their mouse VM counterparts in their preferential trafficking to the liver and their increasing presence in the repertoire during ageing.

The predisposition of CD5hi naive T cells to proliferate in response to antigen or a lymphopenic environment over their CD5lo counterparts is well established^12^,^13^,^14^. It now seems clear that the central determinant favouring the response of CD5hi T cells over CD5lo T cells is not dictated by the TCR specificity or its affinity for cognate antigen. Recent experiments from the Jameson and Allen labs convincingly showed that T-cell responsiveness during antigen challenge was stratified by CD5 expression, even when using a fixed TCR^12^,^14^. Jameson and colleagues proposed that the sensitivity of CD5hi T cells to inflammatory cues facilitated their CD5-stratified response^14^. Our data presented here are in general agreement, but further suggest that sensitivity to IL-15 may be central to this increased responsiveness. This sensitivity may be related to more than just expression of receptor components. For example, Surh and colleagues suggested that an increased sensitivity to IL-2/15 was due to the incorporation of their receptors into lipid raft microdomains^40^. That said, as we have shown here (Fig. 4a–c), there are indeed changes in the expression of gene products necessary for transmission of cytokine signals (*Il2rb*, *Eomes* and *Il4r*), which progressively increase along the CD5lo-memory differentiation continuum (Table 1). However, this is not true for the receptors for IL-7, IL-12 or the common gamma receptor, on which signalling through IL-2, -4, -7 and -15 depends. Thus, the increased efficiency of cytokine signalling in naive CD5hi and VM cells is likely influenced by both progressive expression of signalling components and a subcellular organization that is optimized for signal transduction.

These data ultimately support a model in which selection in the thymus on high-affinity ligands enacts a cellular programme that augments responsiveness to cytokines. For CD8+ T cells, a major outcome of the increased sensitivity to IL-15 is the conversion of a subset of CD5hi T cells into VM. Undergoing VM conversion results in the induction of a host of genes critical for sensing and producing inflammation, thereby facilitating the VM cell response against infectious challenges regardless of antigen specificity. This model lends increasing support for the importance of so-called ‘inflammatory' IL-15, produced during the earliest stages of bacterial or viral infection^41^, as being central for mediating maximal function of both naive CD5hi (ref. 14) and memory CD8+ T cells^42^. Thus, thymic selection not only produces T cells capable of response to foreign antigens but it also produces cells optimized for diversifying the peripheral T-cell repertoire (via cytokine responsiveness) regardless of antigen encounter.

Taken more broadly, our data support a new mechanistic understanding of the progressive differentiation from naive to memory T cells. Bevan and colleagues showed that, contradictory to conventional thought, memory T cells are actually less sensitive to antigen, displaying weaker TCR signalling than naive T cells at the same concentration of antigen^43^. However, memory cells are more sensitive to cytokine-mediated stimuli, engaging both proliferative and effector responses at far lower cytokine concentrations than their naive counterparts. Indeed, the efficiency of the memory cell response to homeostatic cytokines is such that they no longer require ‘tonic' stimulation via their TCR and are able to survive in MHC-deficient hosts^44^. This suggests that at each increasing step in the differentiation process (CD5lo/CD5hi/effector/memory/VM), a CD8+ T cell has increasingly wired its response to be based less on TCR-derived signals and more on signals derived from environmental/cytokine cues. The data we present here demonstrate this principle at each progressive stage of T-cell differentiation with CD5lo cells being relatively quiescent, CD5hi cells being responsive for VM conversion and VM cells being fully recruited into a protective response, all dependent on IL-15, and independent of overt TCR-mediated signals.

It has become increasingly clear that memory T cells possess substantial innate-like effector functions that are capable of mediating potent (in some instances nearly sterilizing) antigen non-specific protective immunity^17^,^45^,^46^,^47^,^48^,^49^ As a result, memory T cells can influence not just the response to secondary antigen encounters, but to any response accompanied by inflammatory or homeostatic cytokine induction. As we show here (Fig. 5), mice devoid of memory CD8+ T cells (3KxRag) are far less capable of managing their bacterial burden than mice with a population of antigen non-specific memory CD8+ T cells. Our data on VM cells perfectly recapitulate the bystander protective function observed for antigen-experienced memory T cells^17^, and further add a necessary role for IL-15 in this process. While the combined action of IL-12/18/15 was known to induce CD8+ T-cell GrzB and NKG2D expression^17^,^48^, our data reveal that IL-15 is required to sustain the expression of these molecules *in vivo* during a bacterial challenge (Fig. 5).

Finally, we provide here convincing evidence for a population of T cells that likely represent a human equivalent of VM cells. While it was previously suggested that this subset represented human ‘innate CD8' T cells, the phenotypic and functional similarities between innate CD8 and VM cells (at least in the mouse) allow either conclusion for this population. Despite their phenotypic similarities, each cell is the culmination of very different differentiation processes; innate CD8s depend on PLZF, NKT cells and IL-4 in the thymus, and VM cells depend on IL-15 responsiveness in the periphery^2^,^6^,^7^. Although their development is well established, a location and function for innate CD8 cells outside of the thymus has been difficult to clarify, and little is known of what becomes of these cells within a lymphoreplete, WT host. In contrast, VM cells (i) represent the dominant subset of MP cells within the antigen-inexperienced B6 host^7^; (ii) are present even in the complete absence of all the factors (PLZF, IL-4 and NKT cells) required to produce innate CD8s (refs 6, 7); (iii) do not require any prior antigen exposure evidenced by their presence in germ-free and TCRxRag^−/−^ hosts^5^,^7^; (iv) traffic extensively to the liver (Fig. 6); (v) display a phenotype consistent with high affinity for self/foreign antigens (Figs 1 and 2); (vi) respond to IL-15 by maintaining expression of effector molecules that mediate bystander protective function (Fig. 5); and (vii) increase substantially in their frequency over the course of ageing^39^. While admittedly lacking in definitive proof, the putative human VM equivalent shares many of these qualities in that they are found in cord blood (no/limited prior antigen exposure), demonstrate a phenotype consistent with an higher affinity for self (Fig. 7) and responsiveness to IL-15 (ref. 37), express effector molecules important for bystander protection^15^, and are found in high frequency in the liver and increasing frequency during ageing (Fig. 7). While the formation of these VM cells in human, as well as those in the mouse, do not appear to require antigenic experience, others have shown that a VM phenotype may be acquired from antigen-experienced memory cells following incubation with IL-2/7/15 (ref. 36). Thus, it remains to be determined to what degree the peripheral pool of VM cells, in mouse or human, can be derived from antigen-experienced memory T cells or naive precursors. In addition, more work is needed to establish actual innate protective capacity of these cells in the human in the absence of antigenic stimulation.

Given the collective data discussed above, it seems reasonable to conclude that the host is generally pre-disposed towards favouring the formation of memory T cells, regardless of specificity. This is, perhaps, the context in which to best view the developmental and functional role of VM cells: by promoting the differentiation of the T cells with highest affinity to self-antigens into VM cells, the host acquires a population of effector cells that are preferentially and rapidly responsive to cytokines but less so to TCR stimulation, ultimately enhancing protective immunity while, possibly, reducing the likelihood of autoimmunity. The need for this dual benefit is most logically centred on the elderly where the decline in lymphocyte development means that the protection of the host to novel antigenic challenges must be increasingly dependent on the functional response of memory cells, with or without antigen specificity. The data we present here on the development, gene expression profile, trafficking and function of VM cells (in mouse and human) draw attention to this populous and versatile T-cell subset as a likely mediator of host protection to a spectrum of immunological challenges.

## Methods

### Mice and reagents

Female C57/Bl6 mice, 6–12 weeks old, were purchased from the National Cancer Institute. IL-15^−/−^, RAG^−/−^3k TCR tg, RAG^−/−^ gBT TCR tg, RAG^−/−^ F5 TCR tg and OTI TCR tg mice (all on the C57/Bl6 background) were bred in either the Biological Resource Center at the National Jewish Center for Health or the vivarium at the University of Colorado Anschutz Medical Center. CD5KO mice were a gift from the Raman lab at the University of Alabama. All mice used for experiments were between 8 weeks and 4 months old, and of either sex. Peptides were purchased from Peprotech and R&D Systems. Fluorochrome-conjugated antibodies against CD3, CD4, CD5, CD8a, CD8b, CD44 and CD45.1 (1:400); CD49d, CD122 and NKG2D (1:200); IFNg and Gzmb (1:50) were purchased from Biolegend or eBioscience. MHC class I tetramers against B8R [TSYKFESV], LCMV [KAVYNFATC], OVA [SIINFEKL] and HSV [SSIEFARL] (1:200) were either purchased from Beckman Coulter or produced as previously described^50^,^51^. All strains of Listeria monocytogenes used are of the 10403s parental strain. The recombinant Listeria monocytogenes 10403s expressing OVA (Lm-OVA) was a gift from the Bevan lab at the University of Washington. The recombinant Listeria monocytogenes 10403s internalin-A mutant strain was a gift from the LeFrancois lab at the University of Connecticut. All mouse protocols were approved by the Institutional Animal Care and Use Committees at either National Jewish Health or the University of Colorado.

### Tissue perfusion and processing

For distinguishing between tissue and vascular CD8s, anti-CD8b (0.6 μg) was mixed with 50 μl heparin and 150 μl Hanks' balanced salt solution (HBSS) and injected intravenously Ten minutes later, mice were killed and perfused with PBS through the vena cava until the liver and lungs appeared completely perfused (enlarged and whitish). All tissues were then processed as previously described^51^,^52^.

### Cell sorting and transfers

Mouse spleens were dissociated as previously described^51^. Splenocytes were then negatively selected for CD8s using the StemCell CD8 selection kit (cat. no. 19753A). The negative fraction was then stained with CD5 (PE), CD44 (PerCP-Cy5.5) and CD49d (AlexaFluor 647). Cells were sorted on a BD FACSAria into ‘CD5lo' (CD44lo, lowest 20% expressers of CD5), ‘CD5hi' (CD44lo, highest 20% expressers of CD5) and ‘VM' (CD44hi, CD49dlo) populations. Cells were then resuspended in PBS and injected via tail vein.

### Tetramer/CD3 stain and calculation

Mouse splenocytes were stained for tetramer and pulled down as described previously^7^. Resulting fractions were then stained for various surface molecules and run on a Dako CyAn flow cytometer. Data were analysed on FlowJo 7.6.5. Tetramer:CD3 ratios were obtained by creating the ratio equation under ‘derived parameters', applying the ratio to the appropriate population and subsequently measuring the population for the gMFI of the derived Tet:CD3 ratio.

### Generation of IL-15 complexes

Resuspended IL-15 and IL-15Ra were incubated at a ratio of 2:9 (for example, for 1 μg IL-15c, 0.18 μg IL-15 and 0.82 μg IL-15Ra) for 30 min at 37°. Resulting complexes were then resuspended in PBS and injected via tail vein. Four days later, mice were killed and splenocytes were analysed for percentage of VM cells.

### LM protection assay

Splenocytes were sorted into the appropriate population (as described above), and 2.0 × 10^5^ VM cells were injected into recipient mice via tail vein injection. Twenty-four hours later, mice received an injection of Listeria monocytogenes via tail vein (1.0 × 10^5^ colony forming units (CFU) per mouse for WT and 2.5 × 10^3^ CFU per mouse for IL-15^−/−^). Four days later, mice were killed and splenic CFUs were determined as described previously^50^.

### Oral Lm infection

Splenocytes were sorted into the appropriate population (as described above), and 2.0 × 10^5^ VM cells were injected into recipient mice via tail vein injection. The next day, mice were deprived of food and water for 4 h. They were then isolated into separate cages and given a piece of bread containing 2 × 10^10^ CFU internalin-A mutant Listeria monocytogenes. Mice remained isolated until entire piece of bread was consumed, and subsequently returned to normal housing. Four days later, splenic CFUs were determined as described previously^50^. Small intestines were removed from the mice that were killed, bisected and scraped to clean away mucous. The intestines were then homogenized and CFUs were determined in the same way as the spleen.

### *In vivo* ICCS

Mice were injected with 250 μg of Brefeldin A/mouse (in 500 μl of PBS) 1.5 h before killing. Spleens were then processed and stained as previously described, with the caveat that all steps were completed with buffers containing Brefeldin A.

### *In vitro* cytokine stimulation

WT CD45.1 splenocytes were negatively selected for CD8 T cells using the StemCell enrichment kit (see above). CD45.1 T cells (1 × 10^6^) were then incubated with 1 × 10^7^ splenocytes from either WT CD45.2 or IL-15^−/−^ (CD45.2) mice in 50 U ml^−1^ IL-2±50 ng ml^−1^ of both IL-12/IL-18. Four hours before collection for staining, Brefeldin A was added to the cultures. All CD45.1+ VM cells were analysed by FACS for IFNg and GrzB.

### RNA isolation

WT C57/Bl6 mouse splenocytes were negatively selected for CD8s using the StemCell CD8 selection kit (cat. no. 19753A). The negative fraction was stained with CD5 (PE), CD44 (PerCP-Cy5.5), CD49d (AlexaFluor 647) and CD8 (APC). Cells were sorted on a BD FACSAria through a CD8+ gate, then sorted into ‘CD5lo' (CD44lo, lowest 20% expressers of CD5), ‘CD5hi' (CD44lo, highest 20% expressers of CD5) and ‘VM' (CD44hi, CD49dlo). Resulting populations were processed for total mRNA using a Qiagen RNeasy plus kit (cat. no. 74134).

### Next-generation sequencing of the transcriptome (RNAseq)

The isolated total RNA from the splenocytes was processed for next-generation sequencing library construction as developed in the NJH Genomics Facility for analysis with a Life Technologies (Carlsbad, CA, USA) Ion Proton next-generation sequencing platform. A modified Kapa Biosystems (Wilmington, MA, USA) KAPA Stranded mRNA-Seq kit for whole-transcriptome libraries was used to primarily target all poly-A RNA. Briefly, library construction proceeded from isolation of total RNA species, followed by mRNA (poly-A) isolation, first and second strand cDNA synthesis, Life Technologies Ion adaptor ligation, amplification and bead templating. Once validated, the libraries were sequenced as barcoded-pooled samples on a P1 Ion Proton chip. Sequence reads of 30 or more nucleotides were mapped to the mm10 (GRCm38) assembly of the mouse genome with the STAR RNA-Seq aligner version 2.4.1d (ref. 53) using gene annotation data from Ensembl version 78 (ref. 54). For each gene in the Ensembl 71 annotation, reads were counted using the HTSeq software version 0.6.0 (ref. 55). Statistical comparisons of the gene expression between the cell types were performed with the DESeq2 package version 1.8.1 (ref. 56) and for the R statistical software version 3.2.0 (ref. 57). All sequencing data are stored in the GEO repository and is accessible under the accession number GSE78247.

### Human reagents

Fluorochrome-conjugated antibodies against CD3, CD4, CD5, CD8a, CD27, CD45RA, CD45RO, CD122, Nur77, Eomes, NKG2A, NKG2D, KIR2D and KIR3DL1 were purchased from eBioscience, Biolegend or Miltenyi Biotec.

### Human cell sources and methods

Human peripheral blood mononuclear cells (PBMCs) were obtained from apheresis LRS chambers that were left over after blood donation. Adult PBMCs were obtained from nine patients, six male and three female, ranging in age from 29 to72 (mean 50) years. Samples were washed with PBS several times and aliquoted before staining. Cord blood samples were obtained from the University of Colorado Cord Blood bank. Samples were diluted 1:1 and run on a Ficoll gradient to isolate the mononuclear layer, which was then isolated and washed. Human spleen and liver samples were obtained from the surgical banks of Drs McCarter and Rosen, respectively. Spleen samples were obtained from surgeries unrelated to haematological malignancies (*n*=7 benign pancreatic mass and *n*=2 non-functioning neuroendocrine tumour), and were determined by a pathologist to be histologically ‘normal'. Healthy liver biopsies were obtained from five normal patients, four males and one female, age ranging from 23 to 56 (mean 40) years. Samples (stored in single-cell suspensions) were thawed, plated, allowed to equilibrate over a few hours in media, washed and stained. All human samples were de-identified by the collecting body before our analysis.

## Additional information

**How to cite this article:** White, J. T. *et al*. Virtual memory T cells develop and mediate bystander protective immunity in an IL-15-dependent manner. *Nat. Commun.* 7:11291 doi: 10.1038/ncomms11291 (2016).

## Supplementary Material

Supplementary Figures Supplementary Figures 1-3

Supplementary Data 1 A master list of all genes identified by RNAseq of sorted, polyclonal CD5lo, CD5hi and VM cells from unmaniupulated B6 mice.

Supplementary Data 2 A comparison of gene expression (RNAseq) between CD5lo and CD5hi naive CD8+ T cells from unmaniupulated B6 mice.

Supplementary Data 3 A comparison of gene expression (RNAseq) between naive CD5hi and VM CD8+ T cells from unmaniupulated B6 mice.

## Figures and Tables

**Table 1:** RNAseq data for cytokine-cytokine receptor KEGG pathway and relevant genes across VM and naive CD5lo/hi.

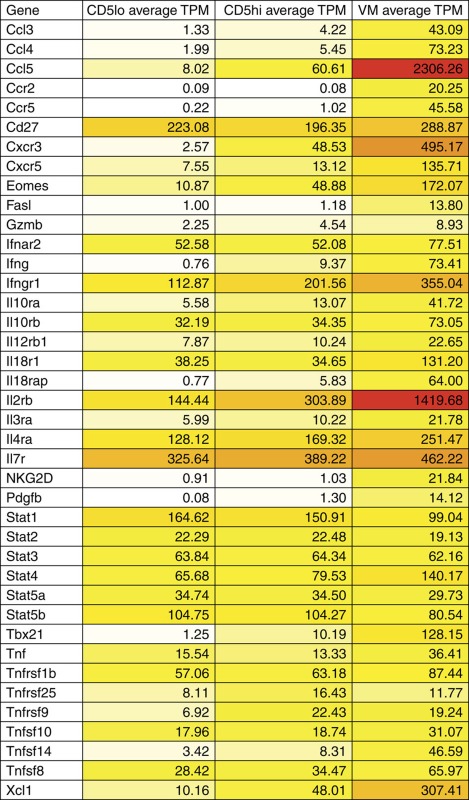

RNAseq, RNA sequencing; TPM, transcripts per million; VM, virtual memory.

Darker shading indicates increased absolute gene expression.

**Table 2:** GO annotations associated with genes upregulated in both CD5 compared with CD5lo and VM compared with CD5hi.

**Gene ontology annotation: CD5lo<CD5hi**	**Bayes factor**
GO:0050877 [4]: neurophysiological process	20.87
GO:0009581 [5]: detection of external stimulus	18.05
GO:0007600 [5]: sensory perception	17.85
GO:0009605 [4]: response to external stimulus	11.08
GO:0042981 [6]: regulation of apoptosis	6.76
GO:0043067 [5]: regulation of programmed cell death	6.59
GO:0007049 [5]: cell cycle	6.3
GO:0008283 [4]: cell proliferation	6.25
GO:0006915 [6]: apoptosis	6.25
GO:0012501 [5]: programmed cell death	6.02
	
**GO annotation: CD5hi<VM**	**Bayes factor**
GO:0050877 [4]: neurophysiological process	47.67
GO:0009581 [5]: detection of external stimulus	43.54
GO:0007600 [5]: sensory perception	43.07
GO:0007186 [6]: G-protein-coupled receptor protein signalling pathway	32.8
GO:0009605 [4]: response to external stimulus	24.63
GO:0007242 [5]: intracellular signalling cascade	13.97
GO:0008219 [4]: cell death	11.57
GO:0016265 [3]: death	11.07
GO:0006793 [5]: phosphorus metabolism	10.35
GO:0006796 [6]: phosphate metabolism	10.35
GO:0051244 [4]: regulation of cellular physiological process	10.17
GO:0043170 [4]: macromolecule metabolism	9.35
GO:0006468 [8]: protein amino-acid phosphorylation	9.12
GO:0044260 [5]: cellular macromolecule metabolism	8.84
GO:0006464 [7]: protein modification	8.67
GO:0016310 [7]: phosphorylation	8.23
GO:0006915 [6]: apoptosis	8.08
GO:0012501 [5]: programmed cell death	7.68
GO:0050874 [3]: organismal physiological process	7.29
GO:0007166 [5]: cell surface receptor linked signal transduction	7.28
GO:0050794 [3]: regulation of cellular process	6.58
GO:0044267 [6]: cellular protein metabolism	6.32
GO:0042981 [6]: regulation of apoptosis	6.09

GO, gene ontology; VM, visual memory.

**Table 3:** List of integrins and adhesion molecules showing significant differences between the VM and naive pools.

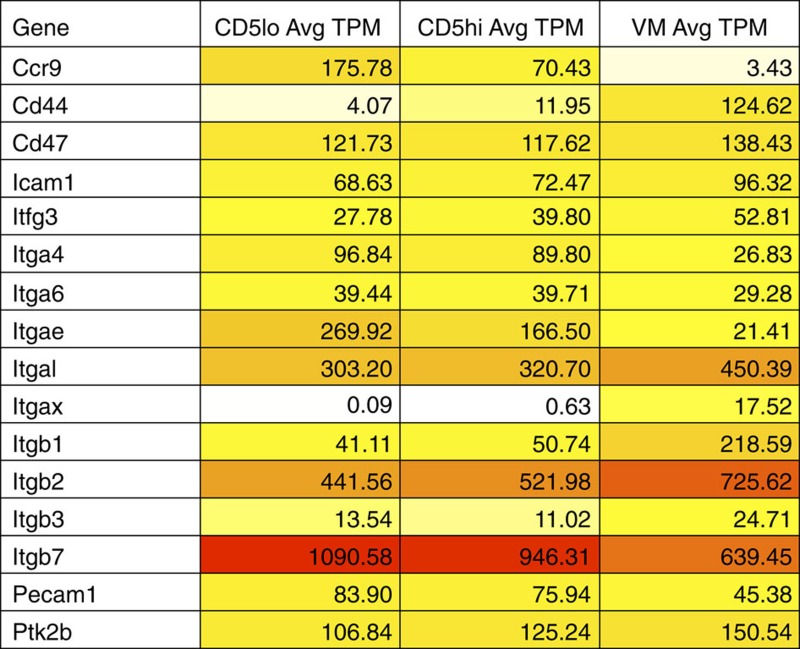

TPM, transcripts per million; VM, virtual memory.

Red values indicate increasing gene expression.
