# Supplementary material for: Virtual memory T cells develop and mediate bystander protective immunity in an IL-15-dependent manner
Source: Nat Commun. 2016 Apr 21;7:11291. doi: 10.1038/ncomms11291 (PMC4844673; doi:10.1038/ncomms11291)
Supplement: Supplementary Figures — 1-3 [file ncomms11291-s1.pdf]

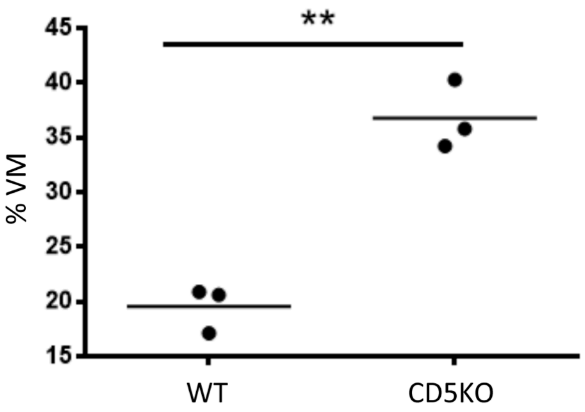

Supplementary Figure 1. CD5KO mice have a higher percentage of VM cells. The percent of VM cells (CD8+CD44hi, CD49dlo) in the spleen were compared between WT and CD5KO mice (**P<0.01, t test. Representative of two experiments with at least n=3/group).

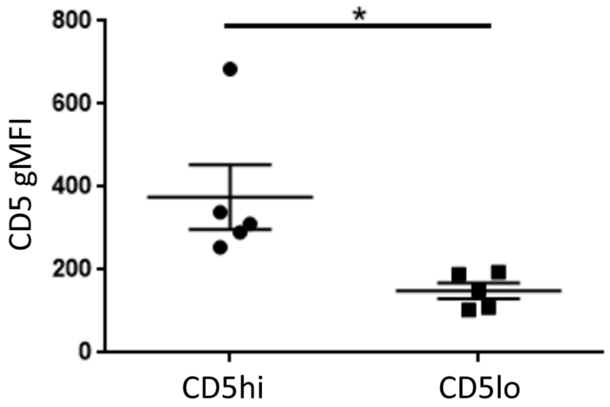

Supplementary Figure 2. CD44loCD5lo (CD5lo) and CD44loCD5hi (CD5hi) cells were adoptively transferred into congenic mice. After three weeks, the mice were sacrificed and the CD5 levels of only the transferred cells which had converted into VM phenotype (CD44hiCD49dlo) were compared. CD5 expression on the allCD5lo-derived VM cells maintained their lower CD5 expression profile relative to the CD5hi-derived VM cells (*P<0.05, t test. Pooled data from two experiments with n=3/group, error bars=SEM).

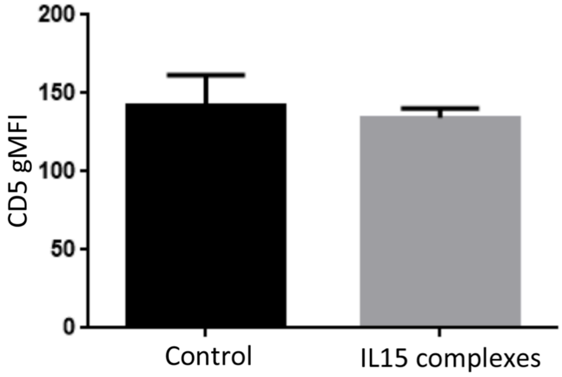

Supplementary Figure 3. OTI VM cells were adoptively transferred into congenic WT mice. 5ug/mouse of IL15 complexes were injected into half of the transferred mice (3 mice per group). Four days later, both injected and control mice were sacrificed and the transferred cells analyzed for their CD5 expression by flow cytometry (error bars=SEM).
